# Laryngeal Schwannoma: A Rare Entity

**DOI:** 10.7759/cureus.31742

**Published:** 2022-11-21

**Authors:** Dema Motter, Basim Wahba

**Affiliations:** 1 Department of Otolaryngology, East Kent Hospitals University NHS Foundation Trust, Kent, GBR; 2 Department of Otolaryngology, Cairo University, Giza, EGY

**Keywords:** benign laryngeal tumour, benign tumour, coblation, neurilemmoma, laryngeal tumour, laryngeal schwannoma

## Abstract

Of all schwannomas, 24-45% arise from the head and neck area. Laryngeal schwannoma is an uncommon site. We report a case of a 34-year-old male who presented to the emergency department with worsening sudden onset inspiratory stridor. On direct laryngoscopy, he was noted to have a laryngeal mass, which caused a ball-and-valve effect on the laryngeal inlet, resulting in airway compromise. He was subsequently intubated and admitted to the intensive therapy unit. He underwent endoscopic removal of a bulky submucosal swelling in the supraglottic region using coblation. The post-operative histopathological assessment confirmed features of laryngeal schwannoma. A six-month post-operative follow-up showed no signs of recurrence. Due to the rarity of this condition, the evidence for the most efficient management of these cases is still not clear. Diagnosis is by histology assessment, although clinical evaluation should raise suspicion. Surgery remains the mainstay of treatment with a good overall prognosis. In current literature, the technique of surgical excision varies with different outcomes. We report a case that was managed with micro-laryngoscopy and coblation with good effect.

## Introduction

Schwannoma is a benign neurogenic tumour that arises from the Schwann cell of the peripheral nervous system. Verocay, in 1908, was the first to describe the tumour [1]. Laryngeal schwannoma is rare and accounts for 0.1-1.5% of all benign neoplasms of the larynx. The vast majority of laryngeal schwannomas arise in the supraglottic area [2]. Due to the limited number of reported cases in the literature, the best treatment modalities, surgical approaches, and the post-operative follow-up required still need further evidence.

## Case presentation

A 34-year-old male presented to the emergency department with a two-hour history of worsening stridor and early respiratory failure. Medical management, including intravenous steroid therapy, adrenaline via a nebulizer (1 mg in 5 ml of normal saline), and high-flow oxygen via a non-rebreather mask, was commenced. After an hour, there was a poor clinical response to the medical therapy, and thus, the decision was taken to intubate the patient with a size 8 endotracheal tube via direct laryngoscopy. The anaesthetist noticed a large laryngeal mass causing a ball-and-valve effect on the laryngeal inlet. The patient had a grade 1 intubation view. The patient had an unremarkable history apart from several months of sensation of a foreign body in the throat. He denied any symptoms of dysphonia, dysphagia, or odynophagia prior to admission. He was a cigarette smoker but otherwise fit and well with no known allergies. Family history was negative for relevant diseases.

He was admitted to the intensive therapy unit (ITU) after being successfully intubated and ventilated. The following day, an attempt to extubate the patient was unsuccessful. He became stridulous, and the decision was taken to re-intubate him. A size 8 percutaneous tracheostomy was then performed to wean the patient off the sedation and ventilation. He was admitted to ITU for eight days, where he was fed via a nasogastric tube.

During his admission, flexible fibreoptic rhino-pharyngo-laryngoscopy revealed the presence of a bulky 60 x 40 mm swelling arising from the right arytenoid cartilage and post-cricoid space. The airway was compromised with the ball-and-valve effect, but both vocal cords were freely mobile. There were no palpable cervical lymph nodes. An endoscopic evaluation has been recorded (Video 1).

**Video 1:** Endoscopic video revealing a large lesion arising from the right arytenoid cartilage with a grade 1 intubation grade.

**Figure 1:**
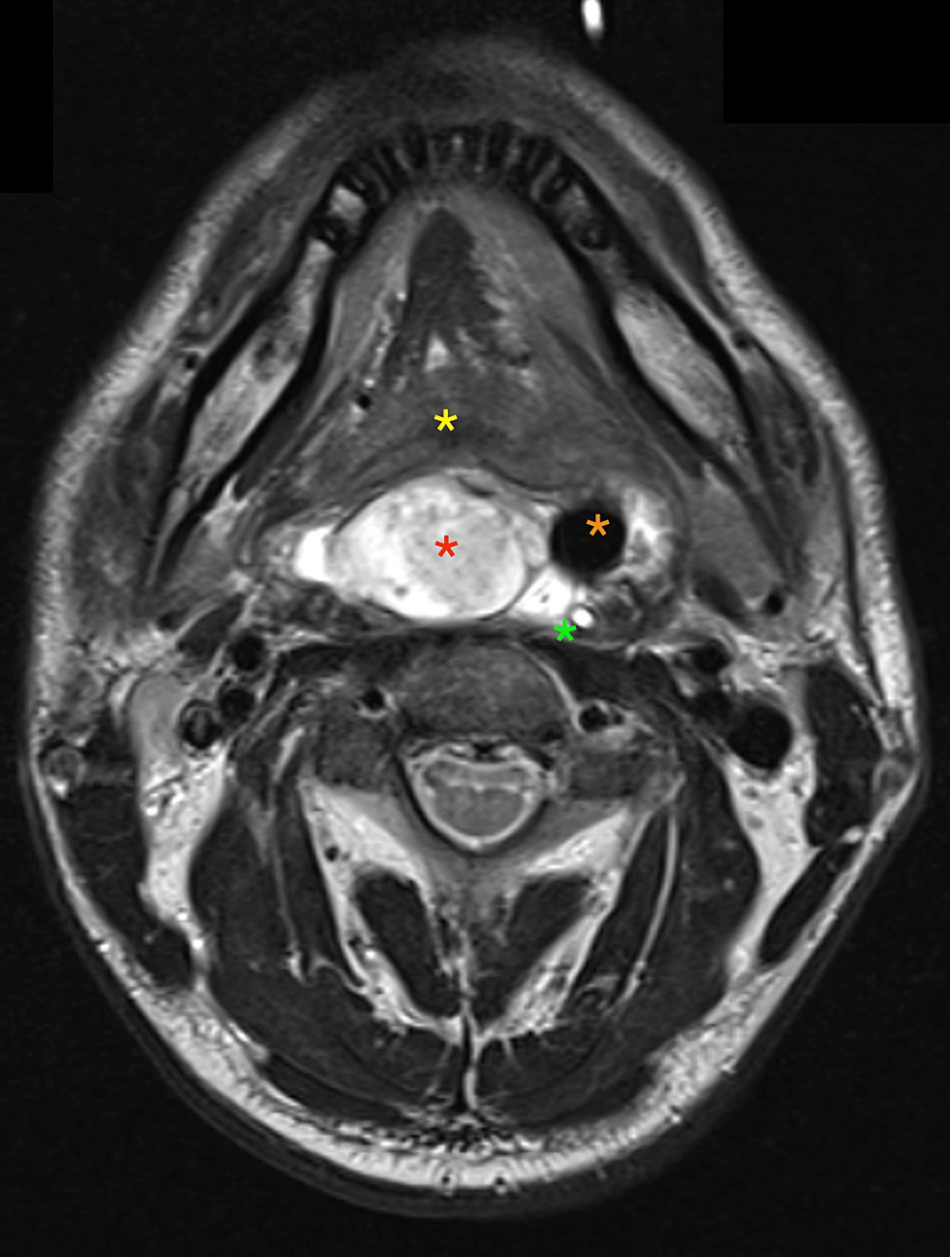
T2-weighted MRI scan of the neck with axial cut showing a large laryngeal mass (red asterisk) occupying the laryngeal inlet and compressing the base of the tongue. The yellow asterisk shows the tongue, the orange asterisk shows the upper airway partially compressed by the mass, and the green asterisk shows the nasogastric tube.

He underwent an MRI scan of the neck, which showed a lobulated, exophytic soft tissue mass occupying the posterior oropharynx/laryngopharynx. It measured approximately 53 mm in craniocaudal diameter and 31 x 20 mm in axial diameter (Figure 1).

**Figure 2:**
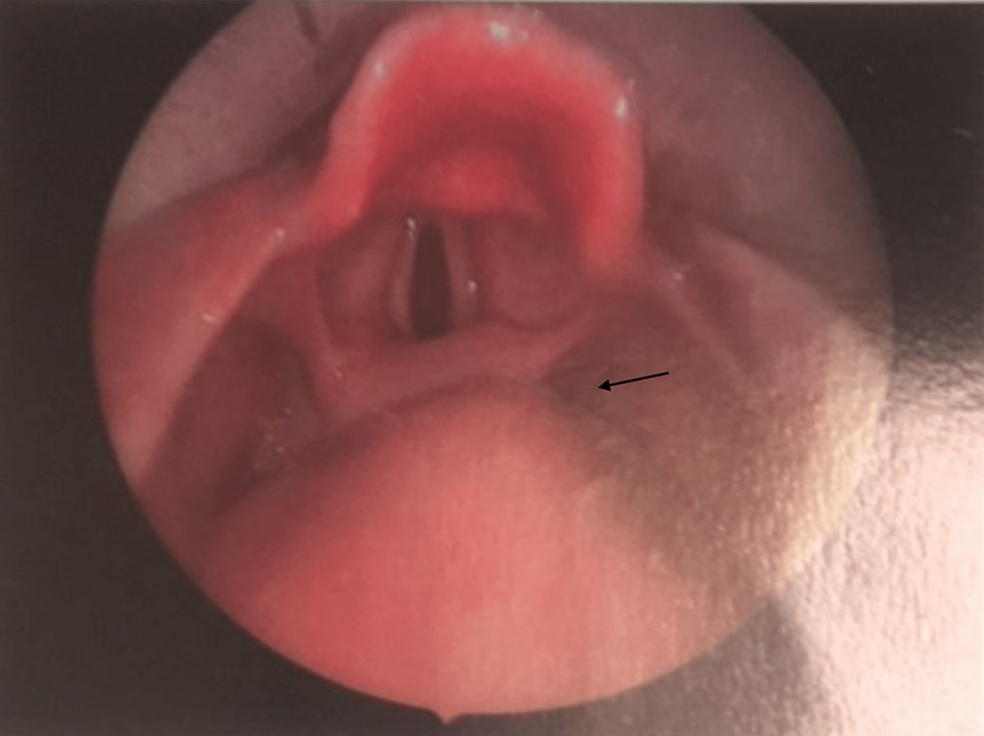
Endoscopic assessment revealing a large lesion arising from the right arytenoid cartilage and post-cricoid space (arrow).

The patient underwent microlaryngosurgery excision of the lesion using a coblation wand. Lindholm laryngoscope was used with a 0-degree Hopkins rod scope. The excision also assisted with a monopolar diathermy laryngeal probe. The surgical specimen was 60 x 40 x 25 mm and weighed 27 grams. The intra-operative photograph is shown in Figure 2.

The following day, the patient reported significant symptomatic improvement, and a repeat flexible fibreoptic nasendoscopy showed bilaterally mobile vocal cords with healing submucosa over the arytenoid cartilage. The tracheostomy was successfully decannulated three days after the procedure. Serial speech and language therapy assessments were performed to improve his swallow function and oral intake.

A histopathological assessment of the specimen confirmed the diagnosis of laryngeal schwannoma. Microscopic examination revealed a roughly circumscribed tumour composed of a proliferation of spindle cells arranged in fascicular patterns with focal palisading and Verocay bodies. There were areas of necrosis. Immunohistochemistry showed that the tumour cells were positive for S-100 protein. CD34 and desmin outline blood vessels only. The features were those of a neurilemmoma (schwannoma). There was no evidence of malignancy.

The patient's symptoms improved significantly, his swallow function returned to normal, and his breathing improved one week after the procedure. He was subsequently discharged home. Follow-up was arranged at six-month intervals for a duration of three years after the operation, and his symptoms completely subsided. Flexible fibreoptic rhino-pharyngo-laryngoscopy did not reveal any signs of recurrence of the disease.

## Discussion

Laryngeal schwannomas (also called neurilemmomas) are encapsulated tumours that arise from benign neoplastic Schwann cells. Schwannoma is a grade I benign tumour, according to the World Health Organization classification of tumours, which describes it as a tumour that resembles normal tissue and is slow growing [3]. It affects patients with a peak incidence between 25 and 50 years of age [4]. Head and neck schwannomas represent about 25-45% of all body schwannomas, with the majority affecting the parapharyngeal space. Laryngeal schwannomas are rare and account for 0.1-1.5% of all benign laryngeal tumours [5,6]. Laryngeal schwannomas have a good overall prognosis if managed appropriately.

Laryngeal schwannoma was first described by Suchanek in 1925 [7]. In Wong et al.'s systematic review of 55 cases of laryngeal schwannoma, they found that the majority of laryngeal schwannomas arise from the supraglottic area, with a significant number arising from the arytenoid cartilage and aryepiglottic fold [8]. It seems that the internal branch of the superior laryngeal nerve is the most frequent nerve of the origin or the plexus between the superior and recurrent laryngeal nerves [2,9].

The clinical presentation depends on the mass size and location of the laryngeal schwannoma. If the mass is small, patients are usually asymptomatic. The severity of symptoms varies and can include non-specific symptoms such as globus sensation, dysphagia/odynophagia, dysphonia, or stridor. Dysphonia may be caused by a decreased mobility of the vocal cords, in the event that the schwannoma originates in them, or more frequently, through impairment of laryngeal mobility by the effect of the tumour mass, which may mimic cricoarytenoid joint fixation [10]. These symptoms can progress over months or years.

Endoscopic evaluation of the larynx will often reveal a submucosal swelling that could contribute to airway compromise. Other imaging modalities, including CT and MRI, are paramount in the diagnosis and management of laryngeal schwannomas. They provide information on the size, location, and extent of the disease. They also have the ability to demonstrate the typical features of a benign disease and aid in surgical planning.

Histopathological assessment is important to distinguish schwannoma from neurofibroma. This is often difficult as the neurofibroma tends to recur, with 10% undergoing malignant transformation. In Enzinger and Weiss's 'Soft Tissue Tumours', they suggest that the histological diagnosis of schwannoma can be made in the presence of three features: (1) clear capsule; (2) presence of Antoni A and B areas; and (3) a positive reaction for S-100 [11].

The literature shows that the main differential diagnoses are benign laryngeal tumours (neurofibroma, lipoma, adenoma, chondroma, papilloma, and paraganglioma) and non-neoplastic lesions (internal laryngocele, ectopic thyroglossal duct cyst, and laryngeal cyst). Malignant lesions should also be excluded. Neurofibromatosis is an important differential diagnosis that should be excluded through careful clinical examination. Clinical features of neurofibromatosis include cafe-au-lait spots, cutaneous nodules, and freckling of skin folds [12]. These features were not present in this patient.

Schwannomas are radioresistant and hence do not respond to radiotherapy [5]. Surgical removal of the mass is the treatment of choice. The surgical approach may be selected depending on the size and site of the tumour. Endoscopic excision can be used if the tumour is small, localized, or pedunculated with good laryngeal exposure. For large tumours, an external approach may provide better exposure for complete excision [6]. Median and lateral thyrotomy are the most frequent external approaches used [8].

In Wong et al.'s systematic review, 53 patients with laryngeal schwannoma underwent surgical excision. Different techniques were observed, including open and transoral approaches [8]. The overall prognosis did not differ depending on the technique used. However, post-operative complications relating to the technique used were not assessed.

Some authors suggest that tumours located in the hypopharynx may not be suitable for endoscopic removal, as poor exposure may result in more mucosal injury [13]. However, in this present case, though the tumour had an origin from the right arytenoid cartilage, we preferred to use an endoscopic approach using the laryngeal coblation wand, which provided a satisfactory haemostatic function compared to the cold steel technique. It also had minimal post-operative pain when compared to diathermy.

## Conclusions

Schwannoma within the larynx is a rare, slow-growing, benign tumour that can present with a variety of symptoms. The overall prognosis for laryngeal schwannoma is good. Schwannomas are radioresistant, and thus surgical excision remains the treatment of choice. We believe that according to the size, site, and extension of the laryngeal schwannoma lesion, the best surgical approach can be determined. Endoscopic coblation using the laryngeal wand is tried for the first time in the treatment of laryngeal schwannoma in this case, and it showed a safe non-invasive way for complete excision of this lesion. A long follow-up is required to ensure that there are no signs of recurrence. Due to the limited number of cases reported in the literature, there is no consensus regarding the best modality of treatment or duration of follow-up required post-operatively.
